# Comparative transcriptome analysis of different tissues of *Rheum tanguticum* Maxim. ex Balf. (Polygonaceae) reveals putative genes involved in anthraquinone biosynthesis

**DOI:** 10.1590/1678-4685-GMB-2021-0407

**Published:** 2022-09-23

**Authors:** Yanping Hu, Huixuan Zhang, Jing Sun, Wenjing Li, Yi Li

**Affiliations:** 1 Qinghai Provincial Key Laboratory of Qinghai-Tibet Plateau Biological Resources, Northwest Institute of Plateau Biology, Chinese Academy of Sciences, Xining, China.; 2 Northwest Institute of Plateau Biology, Chinese Academy of Sciences, Key Laboratory of Adaptation and Evolution of Plateau Biota, Xining, China.; 3 Scientific Research and Popularization Base of Qinghai-Tibet Plateau Biology, Qinghai Provincial Key Laboratory of Animal Ecological Genomics, Xining, China.; 4 University of Chinese Academy of Sciences, Beijing, China.

**Keywords:** Rheum tanguticum, transcriptome, anthraquinone, de novo assembly, secondary metabolism

## Abstract

*Rheum tanguticum* is a perennial herb and an important medicinal plant, with anthraquinones as its main bioactive compounds. However, the specific pathway of anthraquinone biosynthesis in rhubarb is still unclear. The accumulation of anthraquinones in different tissues (root, leaf, stem and seed) of *R. tanguticum* revealed considerable variation, suggesting possible differences in metabolite biosynthetic pathways and accumulation among various tissues. To better illustrate the biosynthetic pathway of anthraquinones, we assembled transcriptome sequences from the root, leaf, stem and seed tissues yielding 157,564 transcripts and 88,142 unigenes. Putative functions could be assigned to 56,911 unigenes (64.57%) based on BLAST searches against annotation databases, including GO, KEGG, Swiss-Prot, NR, and Pfam. In addition, putative genes involved in the biosynthetic pathway of anthraquinone were identified. The expression profiles of nine unigenes involved in anthraquinone biosynthesis were verified in different tissues of *R. tanguticum* by qRT-PCR. Various transcription factors, including bHLH, MYB_related, and C2H2, were identified by searching unigenes against plantTFDB. This is the first transcriptome analysis of different tissues of *R. tanguticum* and can be utilized to describe the genes involved in the biosynthetic pathway of anthraquiones, understanding the molecular mechanism of active compounds in *R. tanguticum*.

## Introduction

*Rheum tanguticum* Maxim. ex Balf., a perennial herb endemic to China, is a species of the *Rheum* genus in the Polygonaceae family (Wu et al., 2003). Dried roots and rhizomes, named rhubarb, have been extensively used as purgative and anti-inflammatory agents in traditional Chinese medicine and Tibetan medicine for 2 000 years (Yang, 1991; Commission, 2015). To date, phytochemical analysis has demonstrated that the major bioactive compounds of *R. tanguticum* are anthraquinones, including emodin, rhein, aloe-emodin, chrysophanol and physcion, which are commonly used as quality markers to assess the medical value of *R. tanguticum* (Cao et al., 2017). These metabolites display various medicinal properties. For example, emodin shows significant antitumor, antimicrobial, antioxidant, and anti-inflammatory properties (Cao *et al.*, 2017; Xia et al., 2019). However, *R. tanguticum* is endemic to China and is only distributed in Qinghai, Gansu and eastern Tibetan Autonomous Region. It required at least three years to obtain a crude drug conforming to the Chinese Pharmacopoeia. As a result of the growth characteristics and heavy exploitation of rhubarb resources, wild resources of *R. tanguticum* are decreasing (Hu et al., 2022). Therefore, it is important to elaborate the transcriptome of *R. tanguticum* and identify candidate genes for the biosynthesis of active constituents to protect this medicinal plant. Anthraquinones are mainly distributed in *R. tanguticum* roots, while only a few anthraquinones are distributed in leaves, stems and seeds by phytochemical analysis (Li, 2010). Therefore, it was reasonable to screen key enzyme genes that catalyze the limiting steps in anthraquinone biosynthesis by comparing the differential expression of candidate genes between roots and other tissues.

Anthraquinones are the main characteristic and pharmacodynamics ingredients of rhubarb. The proportion of anthraquinones ranges from 3 to 5% in different species. More than 30 anthraquinones have been isolated and identified from rhubarb (Gao, 2012). The biosynthetic pathway of anthraquinones was studied in plants of the Rubiaceae family, such as *Morinda*, *Rubia*, and *Ophiorrhiza* species (Inoue et al., 1981; Yamazaki et al., 2013; Veremeichik et al., 2019). It is difficult to elucidate the biosynthetic pathway of anthraquinone because anthraquinone in higher plants is derived from a variety of different precursors and pathways (Reddy et al., 2015). Anthraquinones in plants are derived from a combination of the isochorismate and mevalonic acid (MVA)/2-methyl-d-erythritol-4-phosphate (MEP) pathways (Furumoto and Hoshikuma, 2011; Yamazaki *et al.*, 2013). The backbone of anthraquinone is synthesized by coupling a 1,4-dihydroxy-2-naphthoyl derivative with dimethylallyl diphosphate. However, most of the molecular mechanisms of anthraquinone biosynthesis in *R. tanguticum* remain unknown. 

Next-generation sequencing technologies have been proven to be rapid, cost-effective and efficient means to survey putative genes related to metabolic pathways and imitate the construction of genome and transcriptome databases in non-model species (Egan et al., 2012). RNA-Seq has been used in transcriptome investigations and analysis of differential expression to understand the molecular mechanisms in medicinal plant species without a reference genome sequence, such as *Salvia miltiorrhiza*, *Rubia yunnanensis*, *Saussurea lappa*, and *Digitalis ferruginea* (Bains et al., 2019; Chang et al., 2019; Niu et al., 2020; Yi et al., 2020; Ünlü et al., 2021). 

For *Rheum* species, transcriptome analysis was reported in *R. palmatum* (Li et al., 2018; Liu et al., 2020), *R. officinale* (Hei et al., 2019), and *R. nobile* (Wang et al., 2014), but it has not yet been reported in *R. tanguticum*. In this study, we established transcriptome databases from the root, leaf, stem and seed tissues of over five-year-old *R. tanguticum*. By deep analysis of the transcriptome data, millions of genes, including a series of putative genes underlying anthraquinone biosynthesis and some transcription factors (TFs), were identified. Moreover, qRT-PCR was performed to validate the expression levels of the DEGs involved in anthraquinone biosynthesis. This work sets the foundation for future studies on elaborating the genes participating in anthraquinone biosynthesis, and the molecular mechanism in the biosynthetic pathway of bioactive compounds in *R. tanguticum*.

## Material and Methods

### Plant materials

Three plant materials of over five-year-old *Rheum tanguticum* plants, identified by Dr. Wenjing Li, were collected from Dawu, Maqin County, Golog Prefecture, Qinghai Province, China (34°21′50″ N, 100°29′24″ E, and elevation 3,930 m), in August 2017. Four different parts (root, leaf, stem and seed) were obtained separately from each randomly healthy over five-year-old plant. Therefore, three biological replicates of each tissue were sampled. Part of the tissues were immediately put in liquid nitrogen and stored at -80 ℃ for RNA isolation. Simultaneously, another part of the tissues were harvested and dried at room temperature separately for determination of anthraquinone content.

### Extraction and estimation of anthraquinones

Anthraquinone content was determined by high-performance liquid chromatography (HPLC). Each tissue was dried at room temperature and ground into fine powder. The extraction of anthraquinones was performed according to the Pharmacopoeia of the People’s Republic of China (Commission, 2015). Standard references of anthraquinones (emodin, rhein, aloe-emodin, physcion and chrysophanol) were acquired from the National Institute for the Control of Pharmaceutical and Biological Products (Beijing, China). Each standard substance was melted into methanol to obtain a 0.1 mg/ml solution.

The liquid chromatographic separations were detected by a Thermo Fisher Scientific Ultimate^TM^ 3000 Series HPLC system. The flowing phase was 0.01% methane acid in water (solvent A) and acetonitrile (solvent B). The washed gradient was as follows: 0-4 min, linear from 10-30% B; 4-15 min, linear from 30-48% B; 15-18 min, linear from 48-50% B; 18-19 min, linear from 50-70% B; 19-23 min linear from 70-100% B; 23-30 min, held at 100% B. The flow rate was set as 1.0 ml/min. The sample load was 10 µl. The column temperature was 30 ℃.

The contents of five anthraquinones (emodin, rhein, aloe-emodin, physcion and chrysophanol) in each tissue were analysed by comparing the peak area with those of the respective standards by linear regression. The content was calculated as an average percentage of dry weight (DWT) from three biological replicates.

### RNA extraction and transcriptome sequencing

Total RNA was prepared from each tissue using Total RNA Extractor (Trizol, Sangon Biotech, China) according to the manufacturer’s instructions. The quality and quantity of RNA were detected at the A_260_/A_280_ wavelength ratio with a NanoDrop 2000c spectrophotometer (Thermo Scientific, USA) and RNA integrity was assessed on a 1% denaturing agarose gel to determine the brightness of the 28S and 18S bands. The RNA quality and quantity were further analysed by using a Qubit fluorometer (Invitrogen, USA). Total RNA extracted from different tissues was used to set RNA-Seq paired end sequencing libraries with VAHTS^TM^ mRNA-seq V2 Library Prep Kit for Illumina® (Vazyme Biotech, China). Briefly, this procedure contains mRNA purification, double strand synthesis, end repair, dA tailing, adapter ligation and library amplification.

RNA-Seq libraries were sequenced on the Illumina HiSeq XTen platform (Sangon Biotech, China) by the manufacturer’s instructions, and 150 bp paired-end reads were produced. The raw sequence data studied in this research have been uploaded to the Sequence Read Archive (SRA) at NCBI under BioProject PRJNA608983.

### Data filtering and de novo assembly

The quality of sequencing data was estimated using FastQC (v0.11.2). In addition, adaptors, reads with unknown base calls (N) more than 10%, and low quality sequences (Q < 20) were removed by Trimmomatic (v0.36) to generate clean data. All subsequent analyses were based on clean reads. Clean reads were used for sequence assembly using Trinity (v2.4.0) with min kmer coverage of 2 and other default parameters (Haas et al., 2013). After transcriptome assembly, clean data were mapped to unigenes using Bowtie2 with default parameters (Langmead and Salzberg, 2012). 

### Functional annotation and classification

The assembled sequences were blasted against public databases, including the NR (http://ncbi.nlm.nih.gov/), Swiss-Prot (http://www.expasy.ch/sprot), Clusters of Orthologous Groups (COG) (http://ncbi.nlm.nih.gov/COG/) and Pfam (http://pfam.xfam.org/) databases, using BLAST+ (http://blast.ncbi.nlm.nih.gov/Blast.cgi) with an e-value of 1e^-5^. GO analysis (http://geneontology.org/) was conducted to gain Gene Ontology (GO) functional classifications. NCBI BLAST+ was used to annotate unigenes in Swiss-Prot and TrEMBL databases. Based on these protein annotation results of Swiss-Prot and TrEMBL, GO database was obtained according to the Uniprot’s annotation information. KOG annotation was conducted by NCBI BLAST+ with an e-value of 1e^-5^. KEGG (Kyoto Encyclopedia of Genes and Genomes) classification was performed using the KEGG Automatic Annotation Server (KAAS) with an E value of 1e^-10^. 

### Differentially expressed genes (DEGs)

The transcripts per million (TPM) method was performed to quantify the transcript abundances using Salmon (Patro et al., 2017). Differential expression analysis was conducted using DESeq2 (Anders and Huber, 2010). Genes with a fold change ratio > 2 and qValue < 0.05 were regarded as differentially expressed genes (DEGs). The Venn diagram of DEGs derived from pairwise comparison of different tissues was conducted using VennDiagram R package. ClusterProfiler v3.0.5 (Yu et al., 2012) was used to perform the statistical enrichment of DEGs in KEGG pathways. 

### Transcription factor analysis

Transcription factors (TFs) were predicted by comparing all annotated unigenes against the Plant Transcription Factor Database (PlantTFDB; http://planttfdb.cbi.pku.edu.cn/download.php) using Hmm search with the default settings (Jin et al., 2017).

### Validation of gene expression with qRT-PCR

Quantitative real-time PCR analysis (qRT-PCR) was performed to verify the RNA-seq gene expression profile. Nine unigenes related to anthraquinone biosynthesis were selected for qRT-PCR using the same RNA samples used in RNA-seq. cDNAs were synthesized with total RNA with reverse transcriptase M-MLV (Takara) using oligo d(T)_18_ primers. The primer sequences of these genes are shown in Table S1. qRT-PCR was performed on a LightCycler 480 II Real-Time PCR Cycler (Roche, Switzerland) with SybrGreen qPCR Master Mix (BBI, China). The 18S rRNA of *R. tanguticum* was selected as an internal reference. The 20 µl reaction mixture used contained 2 µl of cDNA, 10 µl of SYBR green qPCR master mix, 0.8 µl of forward and reverse primers and 7.2 µl of ddH_2_O. All reactions were carried out in a LightCycler 480 II Real-Time PCR Cycler (Roche, Switzerland) with the following conditions: an initial step of 3 min at 95 ℃ for, followed by 5 s at 95 ℃ and 30 sat 60 ℃ for 45 cycles. Each reaction was conducted with three biological and three technical repeats. The relative gene expression level for each sample was calculated with the 2^-∆∆Ct^ method (Livak and Schmittgen, 2001). 

## Results

### Anthraquinones accumulation

**Figure 1 -:**
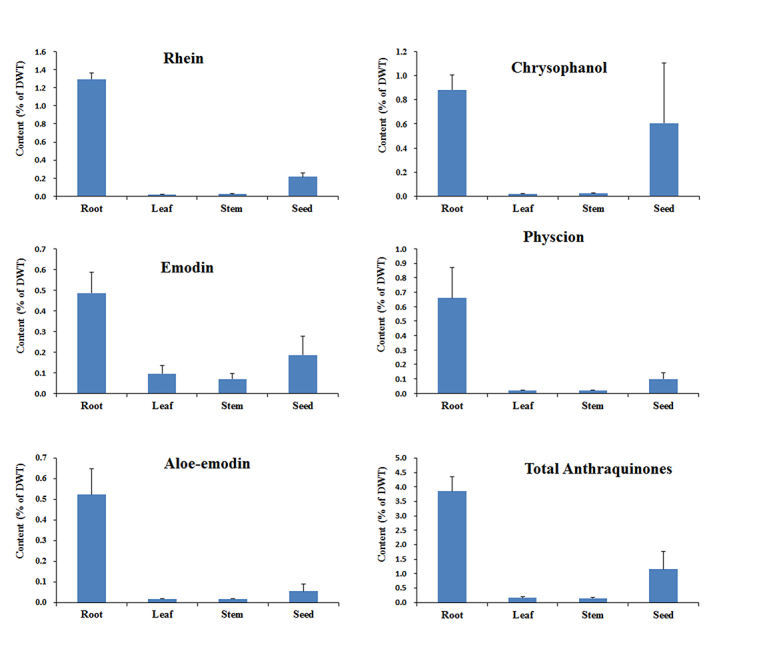
The content of five anthraquines in different tissues of *R. tanguticum*.

To examine the anthraquinones in different parts of *R. tanguticum*, we measured five anthraquinone compounds, emodin, rhein, aloe-emodin, chrysophanol and physcion, in four tissues (root, leaf, stem and seed). The highest contents of emodin, rhein, aloe-emodin, chrysophanol and physcion were all detected in roots (Figure 1), followed by seeds, while the lowest were observed in leaves or stems. The highest accumulation of total anthraquinones was also found in roots, which was as high as 3.85% of DWT. The second highest was in seeds (1.16%), while there was only approximately 0.1% DWT in leaves and stems (Figure 1). As a result of the evident distribution of anthraquinones in various tissues, all four tissues (root, leaf, stem and seed) were used to study the biosynthetic pathway of anthraquinones by comparative transcriptome analysis.

### Transcriptome assembly of *R. tanguticum*

To obtain a transcriptome database of *R. tanguticum*, 12 cDNA libraries were constructed from the four tissues with three biological replicates. All libraries were processed on the Illumina HiSeqXTen platform in paired-end mode. As a result, 546,677,034 clean reads with 79,006,100,895 clean bases were obtained from all of the samples. The averages of Q20 and Q30 were 99.03% and 96.09%, respectively. The average GC content was 60.86% (Table S2). A total of 157,564 transcripts were yielded based on the high-quality reads, with a mean length of 546 bp and 714 bp of N50 length. Finally, 88,142 unigenes were acquired, and the mean length was 514 bp with a N50 equal to 697 bp. Most unigenes were in the size range of 200-500 bp (59,733). There were 17,823 unigenes between 500 bp and 1000 bp, 9264 unigenes of 1000-2000 bp, and 1322 unigenes larger than 2000 bp (Figure S1).

### Functional annotation of *R. tanguticum* unigenes

To obtain a comprehensive annotation profile, the assembled unigenes were blasted against several public databases. The statistical results are shown in Table S3. Out of 88,142 unigenes, 21,331 (24.20%) could be annotated in KOG, 44,771 (50.79%) in GO, 21,862 (24.80%) in Pfam, 40,204 (45.61%) in Swiss-Prot, 47,448 (53.83%) in TrEMBL, 4604 (5.22%) in KEGG, and 48,024 (54.48%) in NR databases. In total, 56,911 unigenes (64.57%) could be annotated to at least one of these databases. The annotated sequences showed significant similarity with those in *Beta vulgaris* subsp. *vulgaris* (6024, 12.55%), followed by *Spinacia oleracea* (3614, 7.53%) and *Vitis vinifera* (3302, 6.88%) (Figure S2).

Gene Ontology (GO) assignments were performed to classify the functions of *R. tanguticum* unigenes. A total of 44,771 unigenes were assigned to three main categories (biological processes, molecular functions, and cellular components). Furthermore, these unigenes were divided into 25 subcategories of biological processes, 22 cellular components and 19 molecular functions (Figure S3). 

**Figure 2-:**
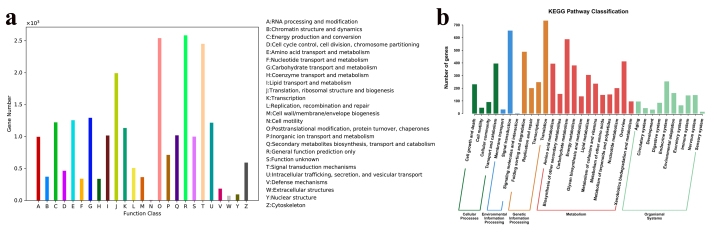
Classification of unigenes and metabolic pathways. a. KOG functional classification of *R. tanguticum* unigenes. b. KEGG classification of metabolic pathways.

Furthermore, the annotated unigenes were classified based on the KOG database for functional prediction. With regard to the KOG database, 21,331 unigenes were assigned to 25 specific functional classes (Figure 2a). General function prediction only (R) was the largest group, followed by posttranslational modification, protein turnover, chaperones (O) and signal transduction mechanisms (T).

The Kyoto Encyclopedia of Genes and Genomes (KEGG) is a useful database worldwide to find potential putative genes in particular biological pathways. The assembled unigenes were mapped to KEGG pathways to understand their function in a certain metabolic pathway in *R. tanguticum* (Figure 2). As a consequence, 4,604 unigenes could be subjected to five main categories: “cellular processes”, “environmental information processing”, “genetic information processing”, “metabolism” and “organismal systems”, with 33 subcategories and 285 pathways (Figure 2b). The most enriched category was “translation” (734), followed by “signal transduction” (655) and “carbohydrate metabolism” (587). Interestingly, there were 669 unigenes in the pathway of secondary metabolite biosynthesis (Table 1). “purine metabolism [PATH: ko00230]” was the largest group (157, 23.47%), followed by “phenylpropanoid biosynthesis [PATH: ko00940]” (82, 12.26%), “terpenoid backbone biosynthesis [PATH: ko00900]” (53, 7.92%), “phenylalanine, tyrosine and tryptophan biosynthesis [PATH: ko00400]” (49, 7.32%), and “porphyrin and chlorophyll metabolism [PATH: ko00860]” (49, 7.32%). Anthraquinone biosynthesis partakes in the isochorismate pathway with phenylpropanoid and partakes in MVA/MEP with sterol and terpenoid (Zheng et al., 2021). Therefore, unigenes that participate in anthraquinone, phenylpropanoid, sterol and terpenoid pathways were reported in the transcriptome of *R. tanguticum* (Table 1). These predicted unigenes involved in the secondary metabolic pathway of the *R. tanguticum* transcriptome will provide rich resources for future research of gene function and gene discovery. 

**Table 1-:** Pathways and number of unigenes related to secondary metabolites in *R. tanguticum.*

Pathway ID	Biosynthesis of secondary metabolites pathway	unigene number
ko00230	Purine metabolism	157
ko00940	Phenylpropanoid biosynthesis	82
ko00900	Terpenoid backbone biosynthesis	53
ko00400	Phenylalanine, tyrosine and tryptophan biosynthesis	49
ko00860	Porphyrin and chlorophyll metabolism	49
ko00130	Ubiquinone and other terpenoid-quinone biosynthesis	34
ko00906	Carotenoid biosynthesis	29
ko00941	Flavonoid biosynthesis	29
ko00100	Steroid biosynthesis	21
ko00904	Diterpenoid biosynthesis	16
ko00950	Isoquinoline alkaloid biosynthesis	16
ko00960	Tropane, piperidine and pyridine alkaloid biosynthesis	16
ko00908	Zeatin biosynthesis	14
ko00909	Sesquiterpenoid and triterpenoid biosynthesis	11
ko00945	Stilbenoid, diarylheptanoid and gingerol biosynthesis	11
ko00521	Streptomycin biosynthesis	10
ko00903	Limonene and pinene degradation	8
ko00905	Brassinosteroid biosynthesis	8
ko00253	Tetracycline biosynthesis	7
ko00261	Monobactam biosynthesis	6
ko00524	Neomycin, kanamycin and gentamicin biosynthesis	6
ko00232	Caffeine metabolism	5
ko00401	Novobiocin biosynthesis	4
ko00942	Anthocyanin biosynthesis	4
ko00965	Betalain biosynthesis	4
ko00254	Aflatoxin biosynthesis	3
ko00332	Carbapenem biosynthesis	3
ko00901	Indole alkaloid biosynthesis	3
ko00944	Flavone and flavonol biosynthesis	3
ko00281	Geraniol degradation	2
ko01051	Biosynthesis of ansamycins	2
ko00523	Polyketide sugar unit biosynthesis	1
ko00902	Monoterpenoid biosynthesis	1
ko00966	Glucosinolate biosynthesis	1
ko01053	Biosynthesis of siderophore group nonribosomal peptides	1

A great variety of TF families, such as MYB, bHLH and WRKY, have been validated to participate in the biosynthesis of secondary metabolites in plants (Yang et al., 2012; Meraj et al., 2020). In the *R. tanguticum* transcriptome analysis, 798 unigenes encoded putative TFs that could be assigned to 47 TF families. The most abundant group was the bHLH TF family (77, 9.65%), followed by the MYB_related (52, 6.52%), C2H2 (51, 6.39%), bZIP (49, 6.14%), ERF (45, 5.64%), NAC (45, 5.64%), C3H (38, 4.76%), WRKY (34, 4.62%), and MYB (33, 4.14%) families (Figure S4). These large numbers of TFs identified in *R. tanguticum* are beneficial to manipulate secondary metabolic mechanisms in this popular medicinal plant.

### Identification and functional analysis of DEGs

**Figure 3 -:**
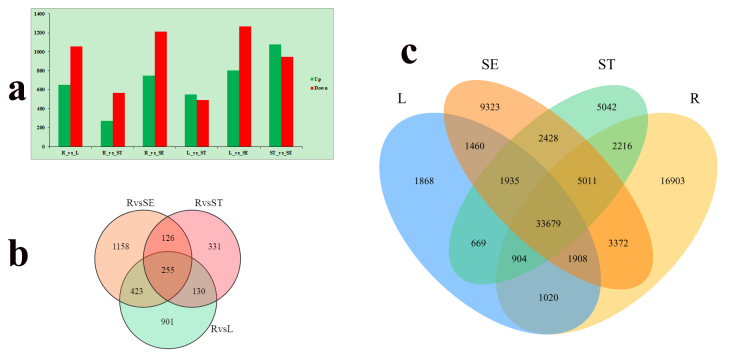
Differential expression analysis of unigenes. a. The number of differentially expressed unigenes (qValue < 0.05 and > 2-fold change) in each tissue compared with other tissues. b. Venn diagram representing the number of DEGs among *R. tanguticum* tissues. c. Coexpression of Venn diagrams among *R. tanguticum* tissues.

DEGs were identified with the DESeq package (fold change ratio > 2 and qValue < 0.05). The highest DEGs were between leaves and seeds (L vs SE, 804 up and 1267 down), and the lowest DEGs were between roots and stems (R vs ST, 274 up and 568 down) (Figure 3a). In comparison groups (R vs L, R vs ST and R vs SE), 255 DEGs were identified in common (Figure 3b). In addition, 33,136 unigenes exhibited tissue-specific expression, with 16,903, 9323, 5042 and 1868 unigenes being specifically expressed in roots, seeds, stems and leaves, respectively (Figure 3c). 

**Figure 4 -:**
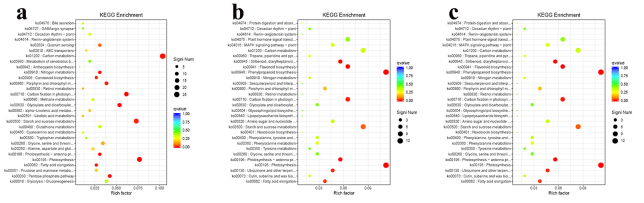
KEGG enrichment analysis of the DEGs among roots, leaves, stems and seeds. a. Between root and leaf, b. between root and stem, c. between root and seed.

All DEGs were subject to KEGG enrichment analysis to identify the possible genes involved in the distinct distribution of anthraquinone. Compared with roots and leaves, carbon metabolism, photosynthesis, carbon fixation in photosynthetic organisms, glyoxylate and dicarboxylate metabolism, photosynthesis-antenna proteins, pentose phosphate pathway, nitrogen metabolism and fatty acid elongation, were the first eight pathways enriched in DEGs (Figure 4a). Between roots and stems, the eight significantly enriched pathways were phenylpropanoid biosynthesis, photosynthesis, carbon fixation in photosynthetic organisms, flavonoid biosynthesis, photosynthesis-antenna proteins, stilbenoid, diarylheptanoid and gingerol biosynthesis, ubiquinone and other terpenoid-quinone biosynthesis and fatty acid elongation (Figure 4b). Between roots and seeds, photosynthesis, phenylpropanoid biosynthesis, photosynthesis-antenna protein, carbon fixation in photosynthetic organisms, fatty acid elongation, tryptophan metabolism, galactose metabolism and cutin, suberine and wax biosynthesis were the eight pathways with significant enrichment (Figure 4c). The secondary metabolic pathways with the highest DEGs, including phenylpropanoid and flavonoid biosynthesis, were determined among various tissues, particularly between roots and aerial parts.

### Putative genes related to anthraquinone biosynthesis

**Figure 5-:**
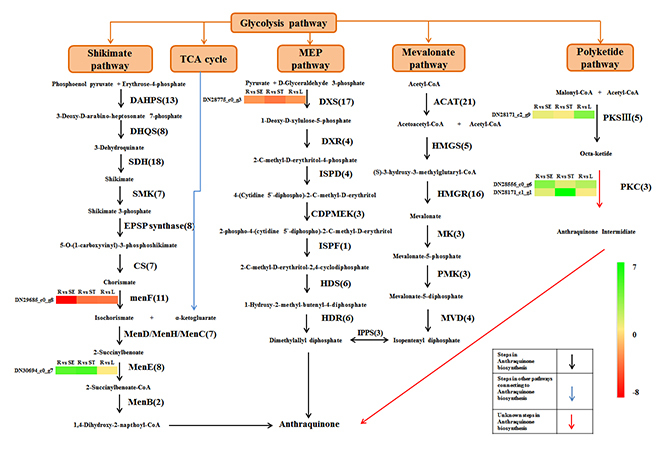
Plausible biosynthetic pathway and unigenes involved in the biosynthesis of anthraquinone in *R. tanguticum*. Note: DXS/DXPS, 1-deoxy-D-xylulose-5-phosphate synthase; DXR, 1-deoxy-D-xylulose-5-phosphate reductoisomerase; ISPD, 2-C-methyl-D-erythritol 4-phosphate cytidylyltransferase; CDPMEK, 4-diphosphocytidyl-2-C-methyl-D-erythritol kinase; ISPF, 2-C-methyl-D-erythritol 2,4-cyclodiphosphate synthase; HDS, (E)-4-hydroxy-3-methylbut-2-enyl-diphosphate synthase; HDR, 4-hydroxy-3-methylbut-2-enyl diphosphatereductase; IPPS, Isopentenyl-diphosphate delta-isomerase; DAHPS, 3-deoxy-7-phosphoheptulonate synthase; DHQS, 3-dehydroquinate synthase; SDH, shikimate dehydrogenase; SMK, Shikimate kinase; EPSP, 3-phosphoshikimate 1-carboxyvinyltransferase; CS, Chorismate synthase; ICS/menF, isochorismate synthase; menD, 2-succinyl-5-enolpyruvyl-6-hydroxy-3-cyclohexene-1-carboxylate synthase; menH, 2-succinyl-6-hydroxy-2,4-cyclohexadiene-1-carboxylate synthase; menC, O-succinylbenzoate synthase; menE, O-succinylbenzoate-CoA ligase; menB, Naphthoate synthase; ACAT, Acetyle-CoA C-acetyltransferase; HMGS, Hydroxymethylglutaryl-CoA synthase; HMGR, Hydroxymethylglutaryl-CoA reductase; MK, Mevalonate kinase; PMK, Phosphomevalonate kinase; MVD, Diphosphomevalonate decarboxylase; PKS Ш, Polyketide synthase Ш; PKC, Polyketide cyclase; The unigenes number of each gene is presented in the brackets.

The anthraquinone biosynthetic pathway partakes in the isochorismate pathway with phenylpropanoid and partakes in MVA/MEP with sterol and terpenoids (Zheng et al., 2021). Dimethylallyl diphosphate, which is the precursor of the anthraquinone backbone, is produced by the MVA/MEP pathways. As a result, we reported the unigenes involved in these pathways of different tissues of *R. tanguticum*. There were 44, 23, 27 and 32 unigenes encoding six enzymes of the MVA pathway in the root, leaf, stem and seed libraries, respectively. There were 31, 17, 29 and 24 unigenes encoding eight enzymes of the MEP pathway in the root, leaf, stem and seed libraries, respectively. Another precursor of anthraquinone backbone 1, 4-dihydroxy-2-napthoyl-CoA, is formed from the substrate of isochorismate produced by the isochorismate pathway. We reported 42, 27, 29 and 27 unigenes encoding six enzymes of the shikimate pathway in the root, leaf, stem and seed libraries, respectively. Similarly, for six enzymes in the menoquinone pathway, 21, 13, 18 and 15 unigenes were identified in the root, leaf, stem and seed libraries, respectively. Moreover, anthraquinone is thought to be synthesized from acetyl-CoA and malonyl-CoA by the polyketide pathway. Polyketide synthase Ш is a key limiting enzyme of the polyketide pathway. In this research, there were 25, 27, 25 and 29 unigenes encoding enzymes in the polyketide pathway in the root, leaf, stem and seed libraries, respectively. CYP450s, SAM-dependent methyltransferases and UDPG are used primarily to modify the anthraquinone backbone to generate various anthraquinone derivatives (Rai et al., 2015). CYP450s catalyze many the oxidative reactions, such as epoxidation, dehydration, hydroxylation and dealkylation. In secondary metabolic biosynthesis such as phenylpropanoids, sterols and terpenoids, CYP450s are members of binding hemoproteins. We found 203, 190, 203, and 293 CYP450s in roots, leaves, stems and seeds encoding CYP450 monooxygenase, CYP450, and NADPH-CYP450 reductase, respectively. With the help of the methyl group of SAM, SAM-dependent methyltransferases catalyze the methylation of the hydroxyl group to produce methyl secondary metabolites (Yoshihara et al., 2008). For SAM-dependent methyltransferases, 21, 19, 21 and 19 unigenes were identified in root, leaf, stem and seed libraries, respectively. Glycosylation generally occurs at the final step of secondary metabolite biosynthesis and results in increased structural diversity, water solubility, and biological activities. UDPG catalyzes the transfer of a sugar from a glycosyl donor to acceptor molecules, thus forming a variety of glycosylated compounds. In our research, we reported 131, 124, 127 and 169 UDPGs in root, leaf, stem and seed tissues, respectively (Figure 5, Table S4).

### qRT-PCR verification of candidate genes related to anthraquinone biosynthesis

**Figure 6-:**
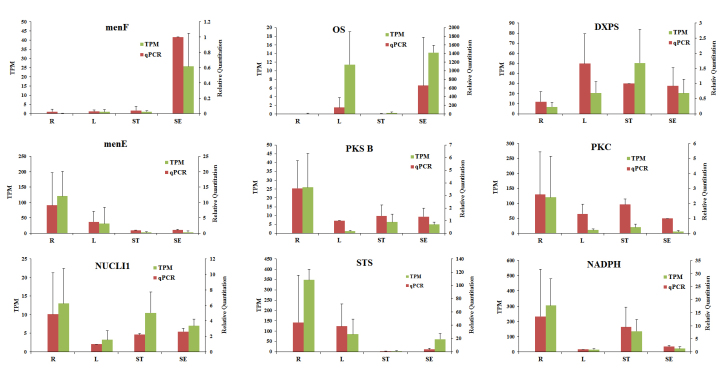
qRT-PCR verification compared with the expression profiles of DEGs (TPM).

To verify the RNA-seq gene expression results, nine unigenes correlated with anthraquinone biosynthesis were selected for qRT-PCR analysis. The results of qRT-PCR and the expression of these unigenes with TPM values are displayed in Figure 6. It was validated that there was a similar expression pattern on account of the TPM value, despite a slight difference in expression folds. *MenF*, *OS* and *DXS* were downregulated in roots compared to other tissues, while *menE*, *PKSB*, *PKC*, *STS*, *NADPH* and *NUCL*1 were upregulated in roots. The qRT-PCR results of these nine unigenes were consistent with the transcriptome analysis. This also confirmed the high degree of reliability of our transcriptome analysis, which will be beneficial for exploring anthraquinone biosynthesis in *R. tanguticum*. 

## Discussion

Although *R. tanguticum* is an ancient and well-known Chinese medicinal plant used worldwide, there are few reports about its functional genomics. Only a few complete or partial gene nucleotide sequences have been deposited in NCBI public databases. No transcriptome or genome information of *R. tanguticum* is available in GenBank. As a consequence, this investigation of transcriptome analysis in *R. tanguticum* is significant for the functional genomics of this medicinal plant. To explore the genes underlying anthraquinone biosynthesis, we performed transcriptome analysis of roots as well as aerial parts (leaves, stems and seeds) with different anthraquinone contents. The anthraquinone content was higher in roots compared with the aerial parts. We assembled four sequenced libraries of roots, leaves, stems and seeds, respectively. In total, 79.01 Gb clean data, and 88,142 unigenes were generated by transcriptome sequencing. The mean length of the unigenes was 514 bp, which is similar to that from *R. nobile* (536 bp). 

Many high-quality reads and fine transcripts acquired from *R. tanguticum* RNA-seq demonstate powerful functionality of *R. tanguticum* sequencing. Species distribution of transcriptomic unigenes against NR database showed that *R. tanguticum* had a closer relationship with *Beta vulgaris*. This result is consistent with the transcriptome in *R. palmatum* (Li et al., 2018) and *R. officinale* (Hei et al., 2019). Out of 88,142 unigenes, 56,911 (64.57%) were aligned to at least one of the NR, KOG, Pfam, KEGG, Swiss-Prot or TrEMBL databases. There were 48,024 unigenes annotated in the NR database, which was 2.5-fold higher than a previous study in the proximal species *R. nobile* (18,501 unigenes) (Wang et al., 2014). The number of the annotated unigenes in the KOG database was 21,331, whereas only 4,560 unigenes were in *R. nobile*. We mapped 4604 unigenes according to the KEGG database, most of which were involved in metabolism. These annotated unigenes based on public databases are useful resources for the study of rhubarb.

Based on large-scale transcriptome data, it is necessary to analyse the putative TF genes related to anthraquinone biosynthesis in *R. tanguticum*. By binding to specific promoter regions, TFs are important regulatory factors in the pathway of plant secondary metabolism (Yang et al., 2012). In our study, nearly 1% (798) of the annotated unigenes was TFs, which were assigned to 47 TF families. The top three TF families in the *R. tanguticum* transcriptome were the bHLH, MYB_related, and C2H2 families. Among all the TF families in *Rheum australe*, bHLH was also the most abundant category followed by MYB related, and NAC (Mala et al., 2021). These transcription factor families known to regulate secondary metabolism play important role in control of anthraquinone biosynthesis (Reddy et al., 2015). Previous reports have shown that bHLH TFs play a key regulatory role in flavonoid and anthocyanin biosynthesis by the pheynylpropanoid pathway (Meraj et al., 2020). MYB is a key TF that negatively regulates the biosynthesis of anthraquinone and seco-iridoid in *Ophiorrhiza pumila* (Rohani et al., 2016). C2H2 is one of the best studied TFs in eukaryotes. It contains one of the best-characterized DNA-binding motifs, which are composed of two Cys and two His residues together with one zinc ion tetrahedrally. C2H2 plays important roles in a variety of biological processes, including DNA/RNA binding, protein interactions and transcriptional regulation (Liu et al., 2022). Therefore, the putative TFs in *R. tanguticum* will supply more adequate resources for regulatory genes in anthraquinone biosynthesis. 

Rhubarb is known around the world for its pharmaceutical anthraquinone; therefore, elucidating the anthraquinone biosynthetic pathway at the transcriptome level is beneficial to increase anthraquinone production through genetic engineering in the near future. By blast against GO and KEGG databases, a large number of unigenes were found in metabolism, genetic information processing, environmental information processing, cellular processes and organismal systems. These unigenes will play important roles in genetic manipulations of rhubarb in the immediate future. The anthraquinone biosynthesis pathway has been reported in the Rubiaceae family such as *Rubia* and *Morinda* plants (Perassolo et al., 2016). In higher plants, it is difficult to elucidate the anthraquinone biosynthetic pathway because anthraquinone compounds are derived from a variety of pathways (Reddy et al., 2015). Generally, anthraquinones are biosynthesized by associating the shikimate pathway (also the isochorismate pathway) and MVA/MEP (Han et al., 2001; Han *et al.*, 2002; Furumoto and Hoshikuma, 2011; Yamazaki et al., 2013; Deng et al., 2018) and through the polyketide pathway (Van den Berg, 1991). The framework of anthraquinones (three benzene rings A, B and C) is produced by the isochorismate and MVA/MEP pathways (Liu et al., 2014). In the initial phase of anthraquinone formation, rings A and B are produced from 1,4-dihydroxy-2-naphthoic acid with two substrates of isochorismic acid and α-ketoglutaric acid. Combined with the MVA/MEP pathway, ring C is biosynthesized from isopentenyl diphosphate (IPP)/3,3-dimethylallyl diphosphate (DMAPP). In this report, most of the genes were detected in the isochorismate and MVA/MEP pathways in the *R. tanguticum* transcriptome (Table S4, Figure 5). In total, 213 structural enzyme genes involved in the isochorismate, MVA, MEP and polyketide pathways were identified, and these genes may regulate the biosynthesis of anthraquinones (Figure 5). Especially, 88 unigenes encoded enzymes involved in the shikimate pathway; 52 unigenes were related to the MVA pathway; 44 unigenes were included in the MEP pathway; 29 unigenes were found in the polyketide pathway. For unigenes with TPM > 10, 17, 10, 10 and 18 unigenes were detected in the isochorimate, MVA, MEP and polyketide pathways, respectively. In the biosynthetic pathway of anthraquinone, RNA-seq was used in *R. palmatum*, *R. officinale* (Liu *et al.*, 2020), *Polygonum cuspidatum* (Zheng et al., 2021), and *Cassia obtusifolia* (Deng *et al.*, 2018). At the early stage of anthraquinone biosynthesis, there are three critical limiting steps, which are catalyzed by IPPS, DXS, and ICS (Liu *et al.*, 2014; Reddy *et al.*, 2015; Deng *et al.*, 2018). Apart from IPPS, DXS, and ICS, we also detected the genes encoding DXR, ISPF, DAHPS, SMK, menE and menH in anthraquinone biosynthesis. To date, few genes (for example, polyketide synthesis from *Rheum emodi*) involved in anthraquinone biosynthesis have been studied in anthraquinone-containing plants. We detected 29 unigenes encoding enzymes in the polyketide pathway. Of these genes, 10 were DEGs in roots comparing with other tissues. Specifically, the expression levels of the four genes including *MenE*, *PKSB*, *PKC* and *STS* were detected significantly upregulated in roots. Meanwhile, we determined the content of five free anthraquinones in different tissues of *R. tanguticum*. Results showed that the contents of five anthraquinones in roots were the highest. Therefore, these four genes may contribute for the accumulation of anthraquinones in the roots. And these steps are probable rate limiting in anthraquinone biosynthesis. Correspondingly, these putative genes might be the key enzymes in the biosynthetic pathway of anthraquinone in *R. tanguticum*. Functional verification of these candidate genes will contribute to revealing the molecular mechanism of active compound biosynthesis, as well as improving the production of these compounds. 

CYPs, SAM-dependent methyltransferases and UDPG are mainly used to modify the anthraquinone backbone to generate various anthraquinone derivatives (Rai et al., 2015). CYPs catalyze many oxidative reactions, such as epoxidation, dehydration, hydroxylation and dealkylation. In secondary metabolic pathways such as phenylpropanoids, alkaloids and terpenoids, CYPs oxidize metabolites with an oxygen atom (Yao et al., 2020; Sharma et al., 2021; Wang et al., 2021). At the final steps of anthraquinone biosynthesis, CYPs modify the framework of anthraquinones (Furumoto and Sato, 2017). With the help of the methyl group of SAM, SAM-dependent methyltransferases catalyze the methylation of the hydroxyl group to produce methyl secondary metabolites (Yoshihara et al., 2008). UDPG catalyzes the transfer of a sugar from a glycosyl donor to acceptor molecules, thus forming a variety of glycosylated metabolites. In the present study, 312, 24 and 176 unigenes in *R. tanguticum* were reported as CYPs, SAM-dependent methyltransferases and UDPGs, respectively. In previous seedling transcriptomes of *R. palmatum* (Li et al., 2018) and *R. officinale* (Hei et al., 2019), only 125 CYP and 73 UDPGs unigenes, and 166 CYP and 66 UDPGs unigenes were reported. The annotated unigene numbers in our research were nearly 2-3 folds greater than the unigene numbers in the other two *Rheum* plants. Therefore, the large number of CYP and UDPG unigenes in our research will lay the foundation for the modifications of anthraquinone derivatives of rhubarb.

## Conclusion

*R. tanguticum* is a famous traditional Chinese medicine endemic to the Qinghai-Tibet Plateau. Anthraquinones are the main pharmaceutical ingredients. To elucidate the biosynthetic pathway of anthraquinone in *R. tanguticum*, four tissues were sampled for transcriptome sequencing. In total, 88,142 unigenes were acquired from twelve RNA-seq libraries of *R. tanguticum*. Candidate genes related to the biosynthetic pathway of anthraquinone were rapidly obtained from this transcriptome analysis. Functional verification of these candidate genes will contribute to revealing the molecular mechanism of active compound biosynthesis, as well as improving the production of these compounds. To the best of our knowledge, this is the first exploration of transcriptome analysis in *R. tanguticum*. Our findings will lay the foundation for the functional genomics of *R. tanguticum*.

## Supplementary material

The following online material is available for this article:

Table S1 - Genes and primers used for qRT-PCR analysis.

Table S2 - Summary of sequencing quality.

Table S3 - Summary of functional annotation of unigenes from BLAST searches against public databases.

Table S4 - Putative genes involved in anthraquinone biosynthesis identified in the leaf, root, seed and stem of *R. tanguticum*.

Figure S1 - Overview of transcriptome assembly showing size distribution.

Figure S2 - Species distribution of the BLASTX results of *R. tanguticum* transcriptome.

Figure S3 - Histogram of gene ontology (GO) classification.

Figure S4 - Distribution of transcription factor families.
