# Supplementary material for: Comparative transcriptome analysis of different tissues of Rheum tanguticum Maxim. ex Balf. (Polygonaceae) reveals putative genes involved in anthraquinone biosynthesis
Source: Genet Mol Biol. 2022 Sep 23;45(3):e20210407. doi: 10.1590/1678-4685-GMB-2021-0407 (PMC9505757; doi:10.1590/1678-4685-GMB-2021-0407)
Supplement: Table S1 - [file 1415-4757-GMB-45-3-e20210407-s1.pdf]

Genetics and Molecular Biology
https://doi.org/10.1590/1678-4685-GMB-2021-0407

Supplementary material to "Comparative transcriptome analysis of different tissues of *Rheum tanguticum* Maxim. ex Balf. (Polygonaceae) reveals putative genes involved in anthraquinone biosynthesis"

Table S1 - Genes and primers used for qRT–PCR analysis.

Gene ID	Name	Amplicon		Sequences
DN28775_c0_g3	DXPS	199 bp	Forward	GCTCGGGGAATGATTAGTGG
			Reverse	CAGCTTTCTCCGCGTATGG
DN29685_c0_g8	menF	222 bp	Forward	GCTTTGTTCTCGCACTCCTT
			Reverse	GCCCTACAGCGGTGGTTT
DN30694_c0_g7	menE	132 bp	Forward	TGGTCCCCGACGTGTAGTTGAG
			Reverse	CTAATCCTCACCGCCAACCCAAC
DN28171_c2_g9	PKS B	133 bp	Forward	GGTGTTGCTGCTTGCGTTT
			Reverse	CGTCTTCTGGGCGGTTCA
DN28556_c0_g6	PKC	107 bp	Forward	AGGAGGTCGCAGCTCTTGA
			Reverse	GCCGTCGTCCAGGAAATC
DN24491_c0_g1	OS	217 bp	Forward	GCCACCTCCAACTACTGCTTC
			Reverse	CACAACCTTCGCCCATAACA
DN26941_c2_g1	NUCLI1	118 bp	Forward	TTTGGAGCAGTCATTGGAGATA
			Reverse	CTGCAAGTGATGTGAAGAAAGG
DN28171_c1_g1	STS	206 bp	Forward	CCCAACAAATCACTCGCACT
			Reverse	TGTTGGTGACACGGAAGTAGAA
DN28182_c2_g2	NADPH	218 bp	Forward	TGCTCATAAGCCCATCTCAAA
			Reverse	GCTGTTAATCAAGTGGAAGT
AB115751.1	18S rRNA	189 bp	Forward	TAGTCCCGTTGCTCTGATGAT
			Reverse	TGGATGTGGTAGCCGTTTCT
