# Supplementary material for: Comparative transcriptome analysis of different tissues of Rheum tanguticum Maxim. ex Balf. (Polygonaceae) reveals putative genes involved in anthraquinone biosynthesis
Source: Genet Mol Biol. 2022 Sep 23;45(3):e20210407. doi: 10.1590/1678-4685-GMB-2021-0407 (PMC9505757; doi:10.1590/1678-4685-GMB-2021-0407)
Supplement: Table S2 - [file 1415-4757-GMB-45-3-e20210407-s2.pdf]

Genetics and Molecular Biology
https://doi.org/10.1590/1678-4685-GMB-2021-0407

Supplementary material to "Comparative transcriptome analysis of different tissues of *Rheum tanguticum* Maxim. ex Balf. (Polygonaceae) reveals putative genes involved in anthraquinone biosynthesis"

Table S2 - Summary of sequencing quality.

	Clean Reads Count	Clean Bases Count (bp)	Clean Bases Gb	Q20 Bases Ratio (%)	Q30 Bases Ratio (%)	GC Bases Ratio (%)
1R	35929654	5208462726	5.208462726	99.06%	96.17%	64.07%
2R	41405146	6000760799	6.000760799	99.04%	96.17%	58.90%
5R	44038600	6350782856	6.350782856	98.97%	95.92%	59.66%
1L	40725840	5888632148	5.888632148	99.03%	96.02%	65.70%
2L	45954734	6644820812	6.644820812	99.01%	95.99%	61.42%
5L	53279000	7725008941	7.725008941	99.06%	96.18%	60.76%
1ST	44945106	6518298262	6.518298262	99.05%	96.12%	62.68%
2ST	48344042	6982465707	6.982465707	98.97%	95.93%	60.22%
5ST	40659614	5873539538	5.873539538	99.01%	96.03%	60.76%
1SE	43341304	6180106075	6.180106075	98.97%	95.94%	60.46%
2SE	56845530	8213752557	8.213752557	99.05%	96.25%	57.37%
5SE	51208464	7419470474	7.419470474	99.08%	96.32%	58.36%
Total	546677034	79006100895	79.0061009			

Note: Q20: percentage of bases with a Phred value >20; Q30: percentage of bases with a Phred value >30; GC (%): Percentage of bases G and C number in the total number of bases.
