# Supplementary material for: Comparative transcriptome analysis of different tissues of Rheum tanguticum Maxim. ex Balf. (Polygonaceae) reveals putative genes involved in anthraquinone biosynthesis
Source: Genet Mol Biol. 2022 Sep 23;45(3):e20210407. doi: 10.1590/1678-4685-GMB-2021-0407 (PMC9505757; doi:10.1590/1678-4685-GMB-2021-0407)
Supplement: Table S3 - [file 1415-4757-GMB-45-3-e20210407-s3.pdf]

Genetics and Molecular Biology
https://doi.org/10.1590/1678-4685-GMB-2021-0407

Supplementary material to "Comparative transcriptome analysis of different tissues of *Rheum tanguticum* Maxim. ex Balf. (Polygonaceae) reveals putative genes involved in anthraquinone biosynthesis"

Table S3 - Summary of functional annotation of unigenes from BLAST searches against public databases.

Annotated databases	Number of genes	Percentage (%)
KOG	21331	24.20
GO	44771	50.79
Pfam	21862	24.80
Swiss-prot	40204	45.61
TrEMBL	47448	53.83
KEGG	4604	5.22
NR	48024	54.48
At least one database	56911	64.57
All database	2303	2.61
Total genes	88142	100
