# Supplementary material for: Comparative transcriptome analysis of different tissues of Rheum tanguticum Maxim. ex Balf. (Polygonaceae) reveals putative genes involved in anthraquinone biosynthesis
Source: Genet Mol Biol. 2022 Sep 23;45(3):e20210407. doi: 10.1590/1678-4685-GMB-2021-0407 (PMC9505757; doi:10.1590/1678-4685-GMB-2021-0407)
Supplement: Table S4 - [file 1415-4757-GMB-45-3-e20210407-s4.pdf]

Genetics and Molecular Biology
https://doi.org/10.1590/1678-4685-GMB-2021-0407

Supplementary material to "Comparative transcriptome analysis of different tissues of *Rheum tanguticum* Maxim. ex Balf. (Polygonaceae) reveals putative genes involved in anthraquinone biosynthesis"

Table S4 - Putative genes involved in anthraquinone biosynthesis identified in the leaf, root, seed and stem of *R. tanguticum*.

Pathway	Gene name	Enzyme symbol	EC	No. of unigenes	No. in leaf	No. in root	No. in seed	No. in stem
MVA 52	Acetyle-CoA C-acetyltransferase	ACAT	2.3.1.9	21	3	17	7	6
	Hydroxymethylglutaryl-CoA synthase	HMGS	2.3.3.10	5	1	4	2	1
	Hydroxymethylglutaryl-CoA reductase	HMGR	1.1.1.34	16	13	15	14	13
	Mevalonate kinase	MK	2.7.1.36	3	3	3	3	3
	Phosphomevalonate kinase	PMK	2.7.4.2	3	2	3	2	2
	Diphosphomevalonate decarboxylase	MVD	4.1.1.33	4	1	2	4	2
total				52	23	44	32	27
MEP 44	1-deoxy-D-xylulose-5-phosphate synthase	DXS	2.2.1.7	17	6	11	12	9
	1-deoxy-D-xylulose-5-phosphate reductoisomerase	DXR	1.1.1.267	4	1	4	1	1
	2-C-methyl-D-erythritol 4-phosphate cytidylyltransferase	ISPD	2.7.7.60	4	2	4	2	3
	4-diphosphocytidyl-2-C-methyl-D-erythritol kinase	CDPMEK	2.7.1.148	3	1	2	1	3
	2-C-methyl-D-erythritol 2,4-cyclodiphosphate synthase	ISPF	4.6.1.12	1	1	1	1	1
	(E)-4-hydroxy-3-methylbut-2-enyl-diphosphate synthase	HDS	1.17.7.1	6	2	4	2	4

	**4-hydroxy-3-methylbut-2-enyl diphosphatereductase**	**HDR**	**1.17.1.2**	**6**	**2**	**3**	**2**	**6**
	**Isopentenyl-diphosphate delta-isomerase**	**IPPS**	**5.3.3.2**	**3**	**2**	**2**	**3**	**2**
total				**44**	**17**	**31**	**24**	**29**
**Shikimate 61**	**3-deoxy-7-phosphoheptulonate synthase**	**DAHPS**	**2.5.1.54**	**13**	**6**	**6**	**7**	**7**
	**3-dehydroquinate synthase**	**DHQS**	**4.2.3.4**	**8**	**4**	**7**	**3**	**4**
	**3-dehydroquinate dehydratase/shikimate dehydrogenase**	**SDH**	**1.1.1.25**	**18**	**7**	**11**	**7**	**10**
	**Shikimate kinase**	**SMK**	**2.7.1.71**	**7**	**6**	**6**	**7**	**6**
	**3-phosphoshikimate 1-carboxyvinyltransferase**	**EPSP**	**2.5.1.19**	**8**	**1**	**7**	**0**	**0**
	**Chorismate synthase**	**CS**	**4.2.3.5**	**7**	**3**	**5**	**3**	**2**
total				**61**	**27**	**42**	**27**	**29**
**Menaquinone pathway 27**	**Menaquinone-specific isochorismate synthase**	**menF**	**5.4.4.2**	**10**	**5**	**7**	**7**	**8**
	**2-succinyl-5-enolpyruvyl-6-hydroxy-3-cyclohexene-1-carboxylate synthase**	**menD**	**2.2.1.9**	**1**	**0**	**0**	**1**	**1**
	**2-succinyl-6-hydroxy-2,4-cyclohexadiene-1-carboxylate synthase**	**menH**	**4.2.99.20**	**5**	**4**	**5**	**4**	**5**
	**O-succinylbenzoate synthase**	**menC**	**4.2.1.113**	**1**	**0**	**1**	**0**	**0**
	**O-succinylbenzoate-CoA ligase**	**menE**	**6.2.1.26**	**8**	**2**	**6**	**1**	**2**
	**Naphthoate synthase**	**menB**	**6.2.1.26**	**2**	**2**	**2**	**2**	**2**
total				**27**	**13**	**21**	**15**	**18**
**Polyketide 29**	**Polyketide synthase III**	**PKS III**	**-**	**4**	**4**	**4**	**4**	**4**
	**Polyketide cyclase**	**PKC**	**-**	**3**	**3**	**3**	**3**	**3**
	**Chalcone synthase**	**CHS**	**2.3.1.74**	**15**	**13**	**11**	**15**	**11**
	**Stilbene synthase**	**STS**	**-**	**3**	**3**	**3**	**3**	**3**
	**Aloesone synthase**	**ALS**	**-**	**2**	**2**	**2**	**2**	**2**
	**Benzalacetone synthase**	**BAS**	**-**	**2**	**2**	**2**	**2**	**2**

https://doi.org/10.1590/1678-4685-GMB-2021-0407

total				**29**	**27**	**25**	**29**	**25**
**Sam dependent methyltransferase**				**24**	**19**	**21**	**19**	**21**
**CYPs 312**	**NADPH-cytochrome P450 reductase**	**-**	**1.6.2.4**	**14**	**8**	**11**	**12**	**9**
	**Cytochrome P450 monooxygenase**	**-**	**1.14.13**	**4**	**3**	**3**	**4**	**2**
	**Cytochrome P450**	**-**	**1.14**	**294**	**179**	**189**	**277**	**192**
total				**312**	**190**	**203**	**293**	**203**
**Glycosylation**	**UDP-glycosyltransferase/UDP -glucosyltransferase**	**UDPG**		**176**	**124**	**131**	**169**	**127**
