# Supplementary material for: Comparative transcriptome analysis of different tissues of Rheum tanguticum Maxim. ex Balf. (Polygonaceae) reveals putative genes involved in anthraquinone biosynthesis
Source: Genet Mol Biol. 2022 Sep 23;45(3):e20210407. doi: 10.1590/1678-4685-GMB-2021-0407 (PMC9505757; doi:10.1590/1678-4685-GMB-2021-0407)
Supplement: Figure S3 - [file 1415-4757-GMB-45-3-e20210407-s7.pdf]

Genetics and Molecular Biology
https://doi.org/10.1590/1678-4685-GMB-2021-0407

Supplementary material to "Comparative transcriptome analysis of different tissues of *Rheum tanguticum* Maxim. ex Balf. (Polygonaceae) reveals putative genes involved in anthraquinone biosynthesis"

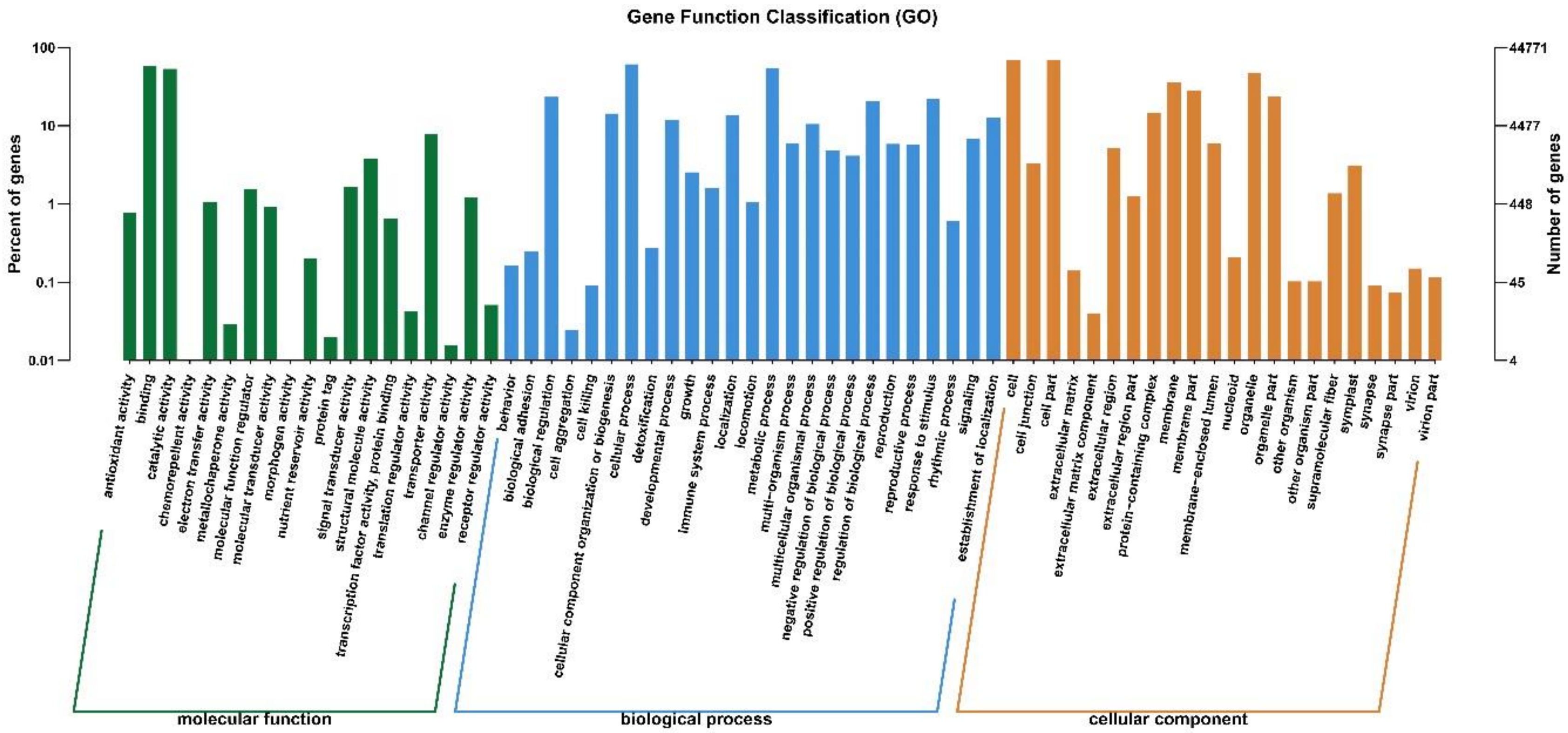

Figure S3 - Histogram of gene ontology (GO) classification. The percentage (left Y-axis) and number (right Y-axis) of unigenes is shown for each category.
